# Chronic exposure of bisphenol S (BPS) affect hypothalamic-pituitary-testicular activities in adult male rats: possible in estrogenic mode of action

**DOI:** 10.1186/s12199-021-00954-0

**Published:** 2021-03-07

**Authors:** Hizb Ullah, Faizan Ullah, Owais Rehman, Sarwat Jahan, Tayyaba Afsar, Dara Al-Disi, Ali Almajwal, Suhail Razak

**Affiliations:** 1 Reproductive Physiology Laboratory, Department of Animal Sciences, Quaid-i-Azam University, Islamabad, Pakistan; 2 University of Science and Technology Bannu, Bannu, Pakistan; 3 Department of Community Health Sciences, College of Applied Medical Sciences, King Saud University, Riyadh, Saudi Arabia

**Keywords:** Bisphenol S, Chronic exposure, Hormonal analysis, Histology, Epididymal sperm

## Abstract

**Background:**

The industrial revolution has resulted in increased synthesis and the introduction of a variety of compounds into the environment and their potentially hazardous effects have been observed in the biota. The present study was aimed to evaluate the potential endocrine-disrupting effects of chronic exposure to the low concentrations of bisphenol S (BPS) in male rats.

**Methods:**

Weaning male Sprague-Dawley rats (22 days old) were either exposed to water containing 0.1% ethanol for control or different concentrations of BPS (0.5, 5, and 50 μg/L) in drinking water for 48 weeks in the chronic exposure study. After completion of the experimental period, animals were dissected and different parameters (hormone concentrations, histology of testis and epididymis, oxidative stress and level of antioxidant enzymes in the testis, daily sperm production (DSP), and sperm parameters) were determined.

**Results:**

Results of the present study showed a significant alteration in the gonadosomatic index (GSI) and relative reproductive organ weights. Oxidative stress in the testis was significantly elevated while sperm motility, daily sperm production, and the number of sperm in epididymis were reduced. Plasma testosterone, luteinizing hormone (LH), and follicle-stimulating hormone (FSH) concentrations were reduced and estradiol levels were high in the 50 μg/L-exposed group. Histological observations involved a significant reduction in the epithelial height of the testis along with disrupted spermatogenesis, an empty lumen of the seminiferous tubules, and the caput region of the epididymis.

**Conclusion:**

These results suggest that exposure to 5 and 50 μg/L of BPS for the chronic duration started from an early age can induce structural changes in testicular tissue architecture and endocrine alterations in the male reproductive system which may lead to infertility in males.

## Background

The increasing population has brought an increased demand and synthesis of various applications for use in day to day life. This demand for application has led the world to the fourth industrial revolution in developed countries. This industrial revolution has resulted in increased synthesis and introduction of a variety of compounds into the environment and their potentially hazardous effects have been observed in the biota [1]. Some of the compounds used in manufacturing plastic, mimic endogenous hormones and can activate/inhibit cellular pathways and are called endocrine-disrupting compounds (EDCs) [2]. BPA has been used in a variety of industrial applications like plastic pipes, baby feeding bottles, and food packaging. Hence, it is present in a huge variety of products to which the common people from newborn babies to adults are exposed. Above 5000 safety-associated investigations have been available on BPA [3]. Owing to its unsafe consequences on physiological and endocrine hormone secretion in living organisms, it has been recognized as leading among EDCs. Further attention has been drawn towards its potential effects on the development and functions of reproductive organs, which depends critically on the hormone. Exposure to BPA for the period of perinatal development of mice has been shown to harm testicular functions at adolescence and maturity [4, 5]. Due to these side effects, there is a ban on its use in many applications throughout Europe and America [6–12] and is replaced by other bisphenols. Bisphenol S is the furthermost considered amongst bisphenol analogs and being the utmost mutual substitute for BPA. The perceptive behind BPS substitution for BPA was less probable to leach monomers into drink and food. This is not an irrational assumption; subsequently, BPS is usually more heat and photo resistant photo as compared to BPA [13]. BPS has been introduced into the industry as a replacement of BPA in several applications and has been considered a “safe alternative” [14, 15]. However, several studies do not support the use of BPS [16] because of its estrogenic and anti-androgenic potential [17, 18]. In the USA and seven Asian countries, BPS concentration, in nanogram to a milligram per gram of receipt papers was reported. It was detected in 81% of urine samples and 0.93 μg/day was the estimated mean daily intake [15, 19]. Liao and Kannan (2012) reported that people occupationally exposed to BPS can absorb 312 ng/kg bw/day through skin, while in the general population the estimated daily intake is 4.18 ng/kg bw/day [15]. In recent studies, different concentrations of BPS have been detected in China and have been correlated with oxidative stress. BPS exposure resulted in unique alterations in the structure of catalase and can affect oxidative stress [20, 21]. Similarly, micromolar concentrations of BPS caused alterations in cell cycle and nuclear morphology in the spermatogonial cells [22]. In female rats, concentrations relevant to a human exposure level of BPS (2 μg/kg) resulted in an alteration in maternal behavior of the rats exposed during pregnancy [23] suggesting that exposure to BPS at very low levels can alter brain functions and behavior.

Exposure of compounds at low concentrations for a prolonged period can cause significant alteration in the physiology and structure of the body organs. Estrogenic compounds are capable of inducing health hazardous and endocrine-disrupting effects in the animals chronically exposed even to very low concentrations of the compound [24]. Low doses effect of BPA has been well documented previously [25] However, very little is known about the effect of chronic BPS exposure in mammals. In a previous lab investigation, it was observed that sub-chronic exposure to different concentrations of BPS resulted in a reduction in the epithelial height of the seminiferous tubules and reduced daily sperm production (DSP) in male rats [26].

The present study aimed to investigate the possible endocrine-disrupting effects of exposure to low concentrations of BPS for a long duration on the male reproductive system using sprague-dawley rats as an animal model. To evaluate the effect on gametogenesis, exposure to low doses of BPS was started at the early stages/age of rat development (approximately 3-week-old rats) and continued for 48 weeks. As the influence of BPS exposure in childhood is of major concern due to bisphenol use in feeding products and its long-term consequences need to be investigated; therefore, we started exposure in weaning age which is equivalent to the childhood phase of humans. As 13.8 rat days are equal to 1 human year. Thus, one human year almost equals two rat weeks (13.8 rat days). whereas because of the different stages of rat lifecycle, comprising weaning to old age, it is conceivable that rats have a short-term childhood phase in comparison to humans. Rats develop rapidly during infancy and become sexually mature at about 6 weeks of age [27]. We studied histopathological alterations at various exposures, biochemical, and hormone analysis to elucidate the effect of chronic BPS exposure on the reproductive health of the adult male.

## Methods

### Animals

Weaning Sprague-Dawley rats (*n* = 40) at postnatal day 22 (PND), weighing approximately 29–32 g were kept under standard laboratory conditions on a 12-h light/dark cycle at 25 °C ± 2 °C. The animals had free access to a standard rat pellet diet with water ad libitum. All animals were obtained from the animal facility of Quaid-i-Azam University Islamabad and maintained following the recommendations of the guidelines for the care and use of laboratory animals (NIH publications no. 80-23; 1996).

### Experimental design

From PND 23, animals were equally divided into four groups. Animals were exposed to different concentrations (0, 0.5, 5, and 50 μg/L BPS mg/kg body weight/day) of BPS for 48 weeks. BPS was dissolved in ethanol which was following published protocols [26, 28], and the stock solution was diluted with water (final concentration of ethanol 0.1%). The duration of the exposure was selected according to the OECD test guideline 452 and the doses and was selected based on a previous study [29]. The BPS solution in the water bottles was daily replaced with fresh solutions. After the completion of the experimental period, animals were weighed, and eight animals/group were killed by cervical dislocation. The number of animals initially recruited for the study was more in consideration of any demise during the prolonged study period. Blood was collected from the heart through a cardiac puncture in heparinized syringes and was subjected to centrifugation at 3000 rpm for 15 min. Plasma was isolated and kept at – 20 ^o^C until hormonal assay. Reproductive organs (testis, epididymis, seminal vesicle, and prostate) were dissected out and weighed for calculation of gonadosomatic index (GSI) and relative weights. Right epididymis and right testis were used for histology while left testis was used for daily sperm production (DSP) and biochemical analysis. Left epididymis was used for the determination of sperm viability, motility, and sperm count in the epididymis.

### Gonadosomatic index (GSI) and relative weight of organs

GSI is an imperative parameter used for the estimation of gonadal maturity in the animals. GSI was obtained for each animal according to the previously described method [29].

$$ \mathrm{GSI}=\frac{\mathrm{Gonadal}\ \mathrm{weight}\ \left(\mathrm{g}\right)}{\mathrm{Body}\ \mathrm{organs}\ \mathrm{weight}\ \left(\mathrm{g}\right)}\times 100 $$

The relative weight of the organs was determined according to the following formula:

$$ \mathrm{Relative}\ \mathrm{weight}\ \left(\frac{\mathrm{mg}}{\mathrm{g}}\right)=\frac{\mathrm{Organ}\ \mathrm{weight}\ \left(\mathrm{mg}\right)}{\mathrm{Body}\ \mathrm{weight}\ \left(\mathrm{g}\right)} $$

Relative weights of the organs were expressed as mg/g body weight.

### Biochemical assays

Tissues collected from various experimental groups were further processed for the antioxidant enzymes. Tissues were homogenized with an automatic homogenizer in phosphate buffer saline (PBS) and centrifuged at 30,000 rpm for 30 min. After the centrifugation, the supernatant was collected and used for the hormonal analysis, protein estimation, and antioxidant enzymes. Quantification of tissue catalase (CAT), *superoxide dismutase (SOD)*, peroxidase (POD), lipid peroxidation (LPO), and reactive oxygen species (ROS) levels were estimated by previously established protocols [30, 31].

### Sperm motility and viability

Immediately after dissection, the cauda epididymis was cut slightly with a scissor in 0.5 mL pre-warmed (at 37 °C) phosphate-buffered saline (pH 7.3) containing a drop of nigrosin stain. An aliquot of 50 μL was taken, placed on a pre-cleaned and warmed (at 37 °C) glass slide, and was observed under a light microscope at × 40. A total of 100 sperm/field were analyzed for motility by a technician blinded to the treatment groups. Each sample was analyzed three times and the average value was used as the total sperm motility. For viability, a drop of eosin and nigrosin was added to the sperm sample. A volume of 10 μL was placed on a pre-warmed and cleaned glass slide and observed under a microscope at × 100. Ten fields were analyzed by a person blinded to the treatment groups. A total of 100 sperm/field were checked for eosin staining and numbers of live and dead sperm were estimated. Each sample was repeated three times and the average number was reported and expressed as a percentage of live sperm [31].

### Tissue histology and morphometry

Testicular tissues (testis and epididymis) were fixed in formalin for 48 h, dehydrated with different grades of alcohol, and cleared with the help of xylene. The paraffin sections (5 μm) were cut and stained with hematoxylin and eosin for histology and morphometry. Transverse sections (10–20/group) of testicular tissues were examined under a Leica microscope (New York Microscope Company) equipped with a digital camera (Canon, Japan).

For the morphometry, the images were taken at × 20 and × 40, and the results were done with ImageJ software. The area of different sections was calculated with previously described protocols [30]. From × 20 images, 30 pictures per animal were selected, and the known area of different areas of intestinal space, epididymis tubules, and seminiferous tubules was measured by the software. The number of different cell types (spermatids, spermatogonia, and spermatocytes) and the area were calculated, and a comparison of different groups with control was done.

### Daily sperm production

DSP was determined according to the method described in Ullah et al. 2017 [32]. Caput/carpus and cauda of the epididymis were cut and weighed. The tissues were cut into small pieces by scissors and were homogenized separately (caput and carpus together and cauda alone) in saline containing 0.5% triton × 100. Sperm suspension was diluted with saline containing nigrosin stain. Twenty microliter sample was loaded into the Neubauer chamber and numbers of sperm in the sample were calculated in different chambers. The numbers of sperm in the sample were obtained by multiplying with dilution factor and numbers of sperm per gram of tissue were reported.

### Hormonal analysis

Plasma testosterone and estrogen were determined by enzymes linked immune sorbent assay (ELISA) kit purchased from Amgenix Inc., USA, while LH and FSH in plasma were determined by ELISA kits purchased from Reddot biotech. Inc. according to the manufacturer’s instructions.

### Statistical analysis

One-way analysis of variance (ANOVA) followed by Dunnet’s multiple comparison test was applied to compare different groups with control using GraphPad Prism 5 software. Values were considered significant at *P* < 0.05.

## Results

### Bodyweight, GSI, absolute, and relative weights of reproductive organs

Mean ± SEM final body weight and organ weights are represented in Table 1. Animals of approximately similar weights were included in the experiments, however, after 48 weeks’ experimental duration, the bodyweight of 50 μg/L BPS-exposed group was significantly high (*P* <0.05) than the control. However, the net weight gain in exposed animals was comparable to control. A significant increase (*P* < 0.05) in final body weight was noted in the exposed groups compared to the control. In the reproductive organs, the testicular weight of the exposed animals was comparable to control; however, GSI of the rats showed a significant (*P* < 0.05) reduction in the group exposed to the highest concentration (50 μg/L) of BPS. Absolute weights of epididymis and prostate were not significantly altered; however, the relative and absolute weight of paired epididymis were significantly reduced in the groups exposed to 5 μg/L (*P* <0.05) and 50 μg/L (*P* < 0.001 and *P* < 0.01, respectively) BPS. The relative weight of the seminal vesicle was also significantly reduced in the highest concentration (50 μg/L) exposed group; however, the prostate weight did not change.

**Table 1:** Effect of chronic exposure of different concentrations of BPS (0.5 μg/L, 5 μg/L, and 50 μg/L) on body and organs weight of male rats

Parameters		Groups		
	Control	0.5 μg/L	5 μg/L	50 μg/L
**Initial body weight (g)**	30.50 ± 0.57	30.00 ± 0.56	30.63 ± 0.53	31.25 ± 0.70
**Final body weight (g)**	537.25 ± 1.19	536.37 ± 2.26	536.13 ± 3.26	545.25 ± 1.51*
**Paired testis (g)**	3.66 ± 0.08	3.59 ± 0.06	3.52 ± 0.03	3.50 ± 0.02
**GSI**	0.68 ± 0.02	0.67 ± 0.01	0.66 ± 0.01	0.64 ± 0.01*
**Absolute paired epididymis weight (g)**	1.42 ± 0.01	1.41 ± 0.01	1.39 ± 0.02	1.36 ± 0.01
**Relative epididymis weight (mg/g)**	2.64 ± 0.02	2.62 ± 0.02	2.60 ± 0.03	2.50 ± 0.02***
**Absolute seminal vesicle weight (g)**	1.89 ± 0.02	1.87 ± 0.02	1.81 ± 0.01*	1.79 ± 0.02**
**Relative seminal vesicle weight (mg/g)**	3.52 ± 0.04	3.48 ± 0.05	3.39 ± 0.05	3.29 ± 0.04**
**Absolute prostate weight (g)**	1.40 ± 0.02	1.43 ± 0.02	1.45 ± 0.02	1.45 ± 0.03
**Relative prostate weight (mg/g)**	2.61 ± 0.04	2.66 ± 0.05	2.69 ± 0.03	2.67 ± 0.06

Values are presented as mean ± SEM (*n* = 8)

*, **, *** indicate significance at *P* < 0.05, *P* < 0.01 and *P* < 0.001 vs control

### Effect of chronic BPS exposure on antioxidant enzymes and oxidative stress biomarkers

Significant reduction in antioxidant enzyme activity and an increase in LPO and ROS were recorded in the testicular tissues after chronic exposure to different concentrations of BPS as presented in Table 2. CAT activity was significantly reduced in BPS 5 μg/L (*P* < 0.05) and 50 μg/L (*P* < 0.001) exposed groups when compared to the control group. Similarly, SOD and POD levels were also considerably (*P* < 0.05 and *P* < 0.01) reduced in the highest concentration (BPS 50 μg/L) exposed group than the control. POD activity was reduced significantly (*P* < 0.05) in the testicular tissues of the animals exposed to 5 μg/L BPS. On the other hand, a significant increase was observed in LPO (*P* < 0.01) and ROS (*P* < 0.01) in the testicular tissues of animals exposed to 50 μg/L BPS for 48 weeks (Table 2).

**Table 2:** Effect of chronic exposure of different concentrations of BPS (0.5 μg/L, 5 μg/L, and 50 μg/L) on oxidative stress in the testicular tissues of male rats

			Parameters		
Groups	CAT (U/mg protein)	SOD (U/mg protein)	POD (U/mg protein)	LPO (U/mg protein)	ROS (U/g tissue)
**Control**	6.17 ± 0.18	31.84 ± 1.08	5.86 ± 0.04	6.97 ± 0.13	97.32 ± 0.77
**BPS 0.5 μg/L**	6.11 ± 0.20	31.26 ± 1.22	5.72 ± 0.10	7.06 ± 0.12	99.14 ± 2.62
**BPS 5 μg/L**	5.58 ± 0.11*	30.48 ± 0.73	5.57 ± 0.09*	7.46 ± 0.19	104.54 ± 2.57
**BPS 50 μg/L**	5.04 ± 0.11***	28.36 ± 0.43*	5.10 ± 0.05**	8.08 ± 0.17**	119.52 ± 3.02**

Values are presented as mean ± SEM (*n* = 8)

*, **,*** indicate significance at *P* < 0.05, *P* < 0.01, and *P* < 0.001 vs control

### Effect of chronic BPS exposure on plasma testosterone, LH, FSH, and estradiol concentrations

Mean ± SEM plasma testosterone (ng/ml) and estradiol concentrations (pg/ml) are presented in Table 3. Significant (*P* < 0.01 and *P* < 0.001, respectively) reduction in plasma testosterone concentrations were observed in the animals exposed to 5 and 50 μg/L BPS for 48 weeks. However, plasma estradiol concentrations in the animals exposed to 5 and 50 μg/L BPS were significantly (*P* < 0.05 and *P* < 0.01, respectively) increased than the control group. No significant difference in the plasma testosterone and estradiol concentrations were noted in the 0.5 μg/L BPS-exposed group when statistically compared with the control. Plasma LH concentration in the treatment groups was found reduced as compared to the control group (Table 4). Significant reduction in plasma LH and FSH levels (*P* < 0.05) was noted in the highest concentration (50 μg/L BPS) exposed group when compared to the control. In other groups, plasma LH and FSH levels were reduced but were not statistically significant.

**Table 3:** Effect of chronic exposure of different concentrations of BPS (0.5 μg/L, 5 μg/L, and 50 μg/L) on plasma testosterone and estradiol concentrations in male rats

		Hormones		
Groups	Testosterone (ng/ml)	Estradiol (pg/ml)	LH (ng/ml)	FSH (mIU/ml)
**Control**	12.36 ± 0.38	2.71 ± 0.24	1.84 ± 0.10	0.79 ± 0.04
**BPS 0.5 μg/L**	11.77 ± 0.37	3.52 ± 0.31	1.54 ± 0.12	0.68 ± 0.09
**BPS 5 μg/L**	10.56 ± 0.52**	3.69 ± 0.32*	1.55 ± 0.05	0.57 ± 0.07
**BPS 50 μg/L**	9.76 ± 0.25***	4.11 ± 0.13**	1.45 ± 0.10*	0.48 ± 0.03*

Values are presented as mean ± SEM (*n* = 8)

*, **, *** indicate significance at *P* < 0.05, *P* < 0.01, and *P* < 0.001 vs control

**Table 4:** Effect of chronic exposure of different concentrations of BPS (0.5 μg/L, 5 μg/L, and 50 μg/L) on sperm parameters and sperm number in epididymis of rats

		Treatments		
Parameters	Control	BPS 0. 5 (μg/L)	BPS 5 (μg/L)	BPS 50 (μg/L)
**Viable sperm (%)**	93.49 ± 1.93	93.23 ± 2.13	92.92 ± 1.16	89.11 ± 1.19
**Motile sperm (%)**	77.87 ± 1.28	78.19 ± 1.77	73.86 ± 2.13	72.93 ± 1.06*
**DSP (× 10**^**6**^**)**	52.03 ± 0.78	50.60 ± 0.89	49.66 ± 0.93	47.26 ± 0.36**
**Caput/carpus epididymis sperm number (× 10**^**6**^**/g organ)**	307.65 ± 6.22	299.76 ± 5.26	287.27 ± 4.54*	287.35 ± 4.10*
**Cauda epididymis sperm number (× 10**^**6**^**/g organ)**	595.23 ± 15.26	597.84 ± 12.99	567.52 ± 13.21	549.05 ± 7.05*

Values are presented as mean ± SEM (*n* = 8)

*, ** indicate significance at *P* < 0.05 and *P* < 0.01 vs control

### Sperm parameters, DSP, and number of sperm in different parts of epididymis after chronic exposure to different concentrations of BPS

Exposure to the highest concentration (50 μg/L) of BPS for 48 weeks caused a significant (*P* < 0.05) reduction in motile sperm percentage but did not show an effect on viable sperm percentage. DPS in the highest concentration (50 μg/L) BPS-exposed group was found significantly (*P* < 0.01) reduced when compared to the control. Similarly, sperm number in caput/corpus epididymis was significantly reduced in the BPS 5 μg/L (*P* < 0.05) and BPS 50 μg/L (*P* < 0.05)-exposed groups, while, sperm number in the cauda epididymis was only significantly reduced (*P* < 0.05) in 50 μg/L BPS-exposed group. However, no significant alterations were observed in the group exposed to 0.5 μg/L BPS (Table 4).

### Histological changes and planimetry of testicular tissue in adult male exposed to different concentrations of BPS for 48 weeks

**Fig. 1:**
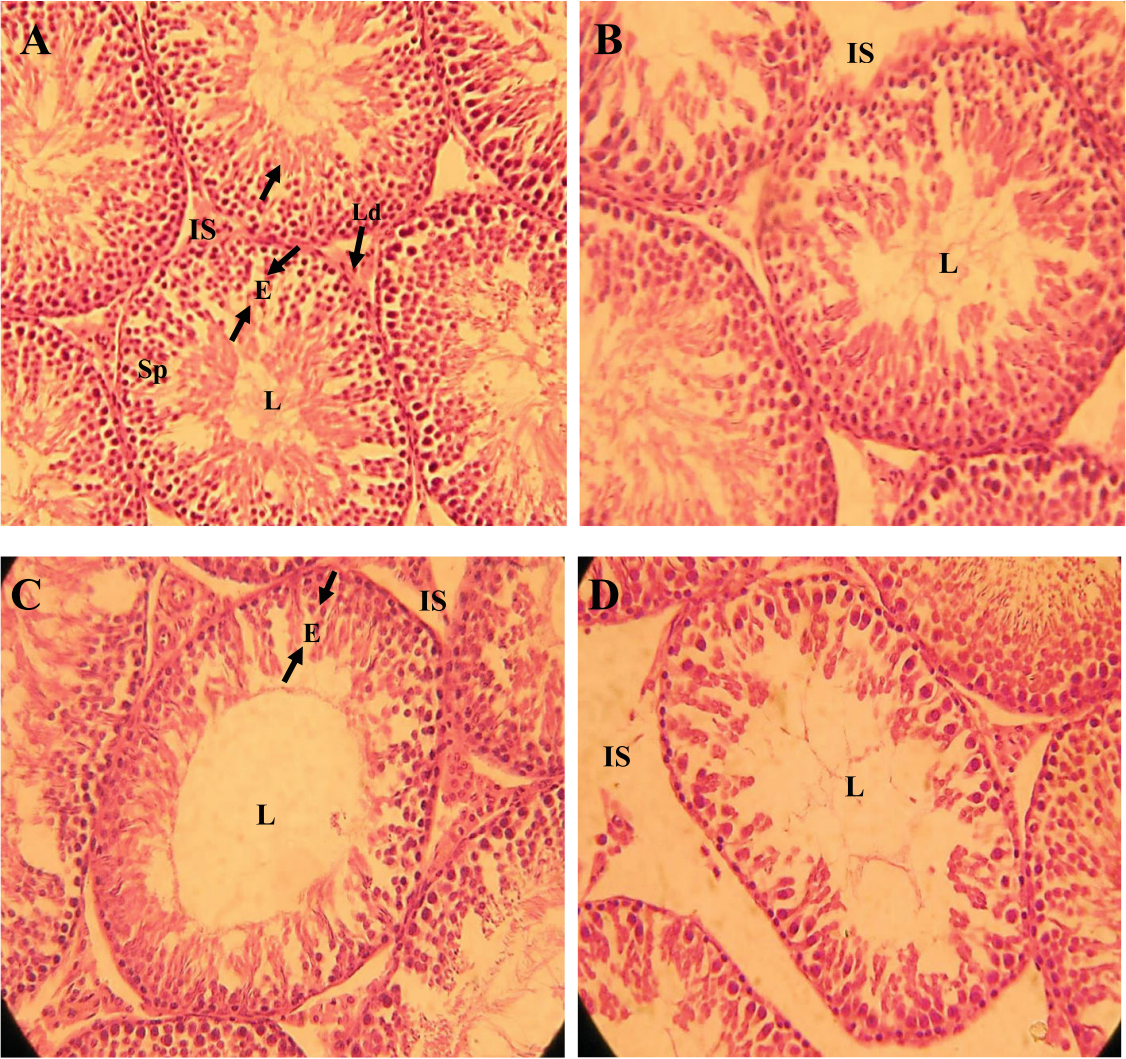
Photomicrograph from testicular tissue showing **a** control; with a compact arrangement of seminiferous tubules having thick epithelium containing normal spermatogonia and sperm-filled lumen (**b**) BPS (0.5 μg/L)-exposed group, presenting normal seminiferous tubules with thick epithelium with different cell types (**c**) 5 μg/L of BPS-exposed group, presenting seminiferous tubules with different cell types and arrested spermatogonia and round spermatids (**d**) 50 μg/L of BPS-exposed group presenting seminiferous tubules with thin epithelium and arrested spermatogonia and elongating spermatids and empty lumen. H&E (× 40)

Histological study of the microscopic slides of the testicular tissues revealed normal morphology of the structures in the control and 0.5 μg/L**-**exposed groups. The seminiferous tubules were compactly arranged with sperm filled lumen and the interstitial space was relatively thin in these groups. In the groups exposed to 5 μg/L and 50 μg/L of BPS, the tubules were relatively alternated with larger interstitial spaces and less filled lumen. Cellular arrest at the spermatogonial stage and round spermatids were more evident in the highest concentration (50 μg/L)-exposed group. In the 5 μg/L-exposed group, a cellular arrest was observed but was less than the 50 μg/L-exposed group (Fig. 1).

Planimetry results showed significant (*P* < 0.5) reduction in the area covered by seminiferous tubules, an area covered by interstitial space, tubular diameter, and epithelial height in the group exposed to 50 μg/L of BPS for 48 weeks. Epithelial height was also significantly reduced in the 5 μg/L-exposed group; however, no alteration was observed in the group exposed to 0.5 μg/L of BPS (Table 5).

**Table 5:** Effect of chronic exposure of different concentrations of BPS (0.5 μg/L, 5 μg/L, and 50 μg/L) on planimetry of testis in rats

Groups				Parameters			
	Area of seminiferous tubules (%)	Area of interstitium (%)	Seminiferous tubule diameter (μm)	Epithelial height (μm)	Spermatogonia (***n***)	Spermatocytes (***n***)	Spermatids (***n***)
Control	84.67 ± 1.62	15.33 ± 1.67	179.96 ± 1.35	73.11 ± 1.55	63.40 ± 1.12	75.48 ± 1.30	258.52 ± 1.92
0.5 μg/L	84.55 ± 1.03	15.45 ± 1.03	179.88 ± 1.65	73.62 ± 1.72	61.80 ± 0.92	75.20 ± 1.35	258.24 ± 2.68
5 μg/L	83.01 ± 1.22	16.99 ± 1.22	176.73 ± 2.05	68.06 ± 0.97*	61.26 ± 1.51	70.08 ± 1.14**	251.36 ± 1.54*
50 μg/L	80.34 ± 0.73*	19.66 ± 0.73*	173.25 ± 2.33*	68.11 ± 0.85*	59.44 ± 1.22	68.32 ± 1.07***	246.32 ± 1.87***

Values are presented as mean ± SEM (*n* = 8)

*, **, *** indicate significance at *P* < 0.05, *P* < 0.01, and *P* < 0.001 vs control

### Number of different cells types in seminiferous tubules of testis of adult rats exposed to different concentrations of BPS for 48 weeks

The number of different cells in the seminiferous tubules of the testis are presented in Table 5. A significant reduction in the number of spermatocytes was observed in the groups exposed to 5 μg/L (*P* < 0.01) and 50 μg/L (*P* < 0.001) than the control group. Similarly, the number of spermatids in the seminiferous tubules were significantly (*P* < 0.05 and *P* < 0.01) reduced in the groups exposed to 5 μg/L of BPS and 50 μg/L, respectively. However, no significant difference was noted in the number of spermatogonia in the exposed groups compared to the control.

### Planimetry and morphological changes in the caput region of the epididymis of rats exposed to different concentrations of BPS for 48 weeks

**Fig. 2:**
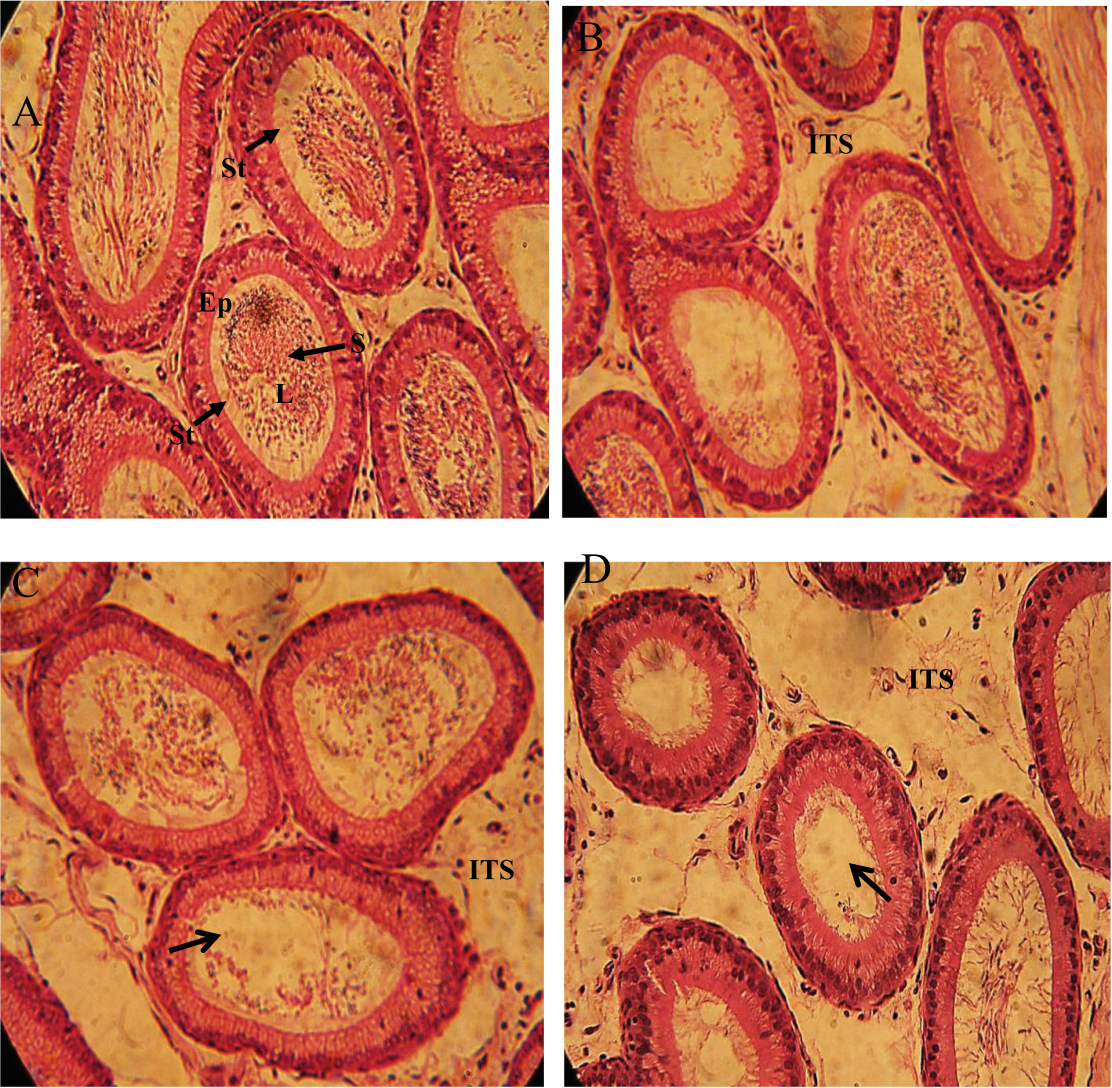
Photomicrograph of caput epididymis tissue showing (**a**) control; with a compact arrangement of caput tubules with sperm filled lumen (**b**) BPS (0.5 μg/L)-exposed group, presenting normal caput tubules like in the control (**c**) BPS (5 μg/L)-exposed group, presenting seminiferous tubules with less number of sperm in the lumen (arrow) (**d**) BPS (50 μg/L)-exposed group presenting caput tubules with an empty lumen (arrow) empty lumen. H&E (× 40)

Planimetry of the caput region of the epididymis showed a significant (*P* < 0.05) reduction in the tubular diameter in the group exposed to 50 μg/L BPS than control after 48 weeks of exposure. No significant difference was noted in other groups exposed to 0.5 and 5 μg/L BPS. Other parameters like lumen diameter, epithelial height, area covered by epithelium, and area covered by lumen did not show any significant alterations compared to the control (Table 6, Fig. 2). The morphological difference observed in the caput region of epididymis showed only a slightly reduced number of sperm in the lumen in the 50 μg/L-exposed group. No significant alterations were evident in other groups in comparison with control (Fig. 2).

**Table 6:** Effect of chronic exposure of different concentrations of BPS (0.5 μg/L, 5 μg/L, and 50 μg/L) on planimetry of caput and cauda epididymis in rats

Groups	Caput epididymis					Cauda epididymis				
	Tubular diameter (μm)	Lumen diameter (μm)	Epithelial height (μm)	Epithelium (%)	Lumen (%)	Tubular diameter (μm)	Lumen diameter (μm)	Epithelial height (μm)	Epithelium (%)	Lumen (%)
Control	361.66 ± 1.55	293.12 ± 3.17	32.12 ± 1.66	31.45 ± 1.44	68.55 ± 1.44	438.13 ± 2.63	411.33 ± 2.88	27.28 ± 1.27	32.16 ± 2.09	67.84 ± 2.08
0.5 μg/L	357.91 ± 2.83	292.34 ± 3.30	33.26 ± 1.49	32.06 ± 1.27	67.95 ± 1.27	439.66 ± 2.93	410.22 ± 3.16	27.11 ± 1.91	31.14 ± 2.18	68.86 ± 2.18
5 μg/L	353.72 ± 3.21	285.23 ± 2.54	29.55 ± 2.14	31.47 ± 1.71	68.53 ± `1.71	433.11 ± 2.67	408.51 ± 2.94	25.34 ± 1.29	30.67 ± 1.88	69.33 ± 1.88
50 μg/L	353.38 ± 1.33*	286.13 ± 2.71	30.31 ± 1.82	31.85 ± 1.46	68.15 ± 1.46	436.21 ± 2.39	407.88 ± 4.36	25.15 ± 1.34	29.33 ± 2.63	70.67 ± 2.63

Values are presented as mean ± SEM (*n* = 8)

* indicate significance at *P* < 0.05 vs control

### Planimetry and morphological changes in the cauda region of the epididymis of rats exposed to different concentrations of BPS for 48 weeks

**Fig. 3:**
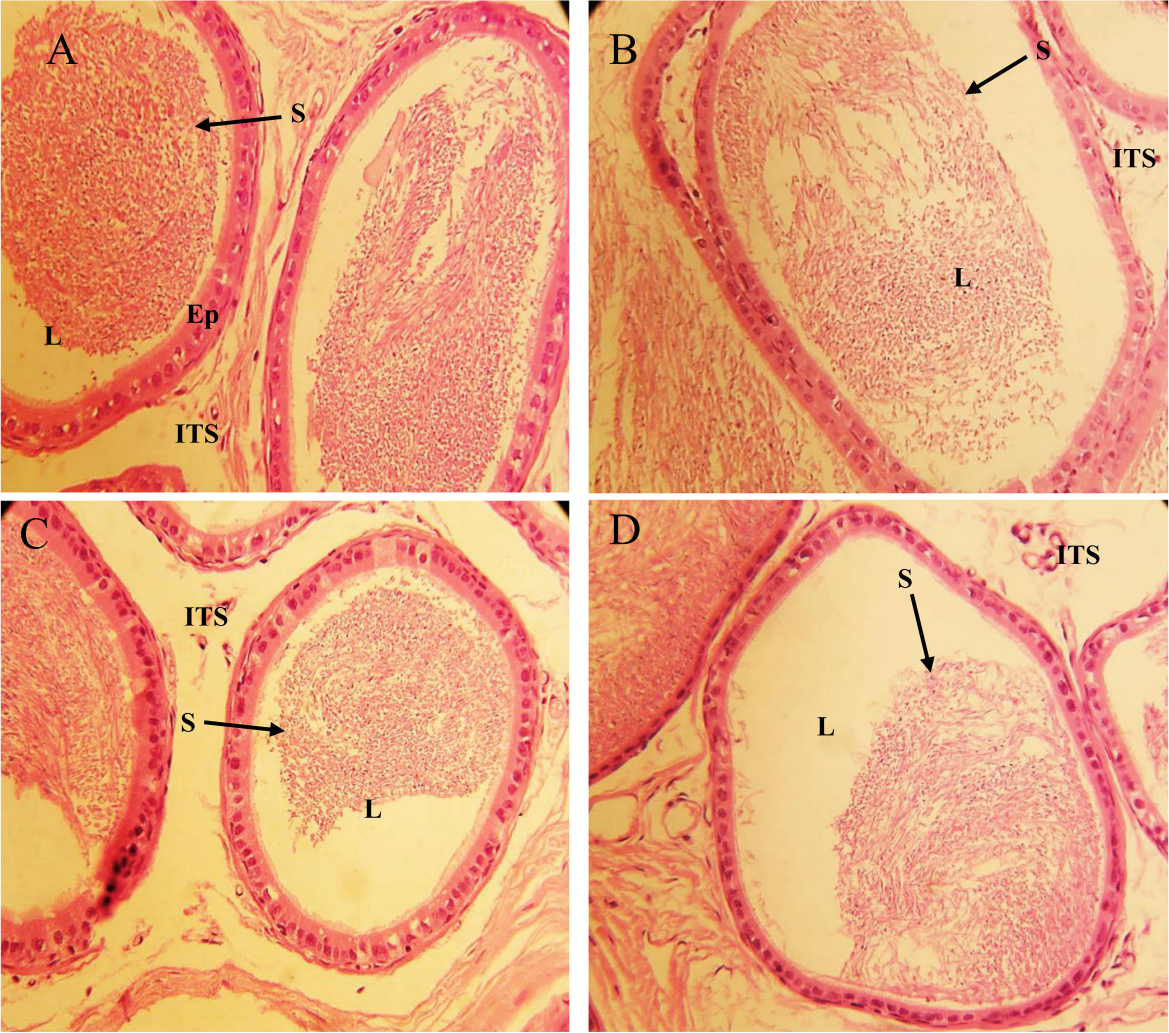
Photomicrograph of cauda epididymis tissue showing (**a**) control; with a compact arrangement of cauda tubules with sperm filled lumen (**b**) BPS (0.5 μg/L)-exposed group, presenting normal caput tubules like in the control (**c**) BPS (5 μg/L)-exposed group, presenting cauda tubules with sperm filled lumen (**d**) BPS (50 μg/L)-exposed group presenting cauda tubules with less sperm in the lumen. H&E (× 40)

Planimetry of the cauda region of the epididymis showed no significant alteration in the tubular diameter in the group exposed to different concentrations of BPS than control after 48 weeks of exposure. Similarly, other parameters like lumen diameter, epithelial height, an area covered by epithelium, and an area covered by lumen did not show any significant alterations compared to the control (Table 6, Fig. 3). The morphological difference observed in the cauda region of epididymis showed only a slightly reduced number of sperm in the lumen in the 50 μg/L-exposed group. No significant alterations were evident in other groups in comparison with control (Fig. 3).

## Discussion

Exposure of living cells under both in vitro and in vivo conditions to BPS is investigated recently and the effects of this compound on living tissues and cells have been manipulated to some extent which is capturing the attention of the researchers in the field of toxicology. The effect of higher concentrations of BPS resulted in uterine growth in female rats [33], altered embryonic viability, and caused an increase in gallbladder size in the chicken embryo [34]. Regarding the low concentrations studies, BPS in femtomolar to nano-molar concentrations activated estrogen receptor alpha-mediated pathways and showed estrogenic and anti-androgenic effects [16]. Similarly, low doses, relevant to human exposure levels, resulted in altered maternal behavior of female rats [23] suggesting the hazardous effect of BPS in mammals. In the present study, male rats were exposed to different concentrations of BPS for 48 weeks. Reduction in GSI, relative weights of reproductive organs, testosterone, LH and FSH concentrations, and alterations in tissue histology were observed in the groups exposed to higher concentrations of BPS. Oxidative stress in the testicular tissue was induced and the DSP was also curtailed in the higher concentration-exposed group than control.

We observed a reduction in plasma LH and FSH concentrations in the high dose group, which in turn contributed to the drop in testosterone levels, while estradiol concentration was high in the groups exposed to higher concentrations of BPS. These findings suggest that BPS has either affected GnRH secretion from the hypothalamus or has directly inhibited gonadotropin secretion from the pituitary which caused a reduction in testosterone which needs to be determined. Similarly, prolonged oxidative stress in the testicular tissues has likely affected the testicular machinery to synthesize testosterone. In previous studies, it was reported that testosterone secretion was suppressed because of P450, which was affected by cisplatin-induced ROS [30, 35]. The oxidative stress-inducing potential of BPS has been documented previously. It was observed that BPS exposure resulted in increased oxidative stress in the peripheral blood mononuclear cells [36] and testis [26]. Oxidative stress in the cells leads to lipids and protein degradation when exposed to BPS in vitro [36]. The inhibition in testosterone secretion is in line with the studies of Rochester and Bolden [16]; they observed the anti-androgenic effect of BPS in vitro. The anti-androgenic effect of BPS was also observed by Molina-Molina et al. (2013) and Kitamura et al. (2005) [37, 38]. The reduced testosterone might be due to the suppression of GnRH transcripts in the hypothalamus as observed by Ji et al. (2013) which suggests that BPS exposure has suppressed GnRH which led to the reduction in gonadotropins secretion [39]. However, the increase in estrogen levels seems to be due to the estrogenic mode of action of BPS.

Besides the reduction in the testosterone concentrations, reduced sperm motility, DSP, and the drop in the number of sperm in epididymis were observed in the high-dose-exposed group as compared to the control. Similarly, GSI and relative weight of the reproductive organs were observed in the highest concentrations of BPS-exposed groups. Recently, exposure to BPS resulted in an increase in adipogenesis in human preadipocytes in vitro [40]. In the mice, BPS exposure during prenatal stages of life resulted in obesity when fed with a high-fat diet [41]. On the other hand, different cell types in the seminiferous tubules were deteriorated along with the lessening in the seminiferous tubule epithelial height. The alterations in these parameters were accompanied by an arrest in spermatogonial cells and round spermatids, which seems to be the cause of the low DSP, the number of sperm in the epididymis, and epithelial height. Correspondingly, reduced GSI has been linked to gonadal maturation [42] and has been associated with poor reproductive functions. In current observation, reduction in the GSI of the male rats after chronic exposures to BPS is linked with the other parameters like truncated daily sperm production and deviations in the seminiferous tubules. In our study, a reduction in the LH and FSH levels supports the histological alterations in the testis and reduction in sperm production. In literature, estrogenic compounds have been well characterized for their effects on a reduction in the weight of reproductive organs in adulthood [43–45]. The reason for the reduction in weight and spermatogenesis is the presence of both estrogen and androgen receptors in these organs that play a crucial role in spermatogenesis. Similarly, gonadotropin receptors play a role in androgen synthesis and spermatogenesis. It has been reported that alteration in these receptors expression can lead to alterations in the testis physiology and spermatogenesis [46–48].

In spermatogonial cells, BPS exposure resulted in alteration in the structure of catalase enzyme and induced toxicity in the cells [21]. Similarly, BPS exposure altered spermatogonial cells' nuclear morphology and affected cell cycle suggesting the potentially hazardous effects of BPS on spermatogenesis [49]. In an in vitro study, BPS exposure induced antioxidant enzyme activity in the sperm and increased ROS concentrations after two hours of exposure caused a reduction in the DSP and altered seminiferous tubule epithelium [26, 50].

## Conclusion

Based on the results from the present study, it can be concluded that exposure for a long period, low concentrations of BPS are capable of suppressing gonadotropins secretion from pituitary, exhibiting estrogenic and anti-androgenic effects in the mammals, induces oxidative stress in the testicular tissue but can affect spermatogenesis by causing arrest at the spermatogonial stage as well as at the stage when spermatids can be seen. Further molecular studies need to be done to identify the exact mechanism of action of BPS through which it exhibits potential hazardous effects in the male reproductive tissues of mammals.

## Data Availability

All the data are contained in the manuscript.
